# Dr. Lester Charles Mitchell

**Published:** 1915-03

**Authors:** 


**Dr. Lester Charles Mitchell,** (not listed in State Directory or

Polk) died at Hamburg, Feb. 12. He was born in Oswego Co. of Colonial stock. He served throughout the Civil War and was a member of Gen. Custer's staff. He studied medicine after the War and practiced medicine in Joliet, Ill. Becoming interested in business, he lived for some years in Minneapolis but joined his family in Hamburg, two years ago. At the time of his death, he was President of the Aberdeen Milling Co., of Aberdeen, S. D.

